# Effect of High-Intensity Interval Training on Aerobic Capacity and Heart Rate Control of Heart Transplant Recipients: a Systematic Review with Meta-Analysis

**DOI:** 10.21470/1678-9741-2019-0420

**Published:** 2021

**Authors:** Lino Sergio Rocha Conceição, Caroline Oliveira Gois, Raiane Eunice Santos Fernandes, Paulo Ricardo Saquete Martins-Filho, Mansueto Gomes Neto, Victor Ribeiro Neves, Vitor Oliveira Carvalho

**Affiliations:** 1 Department of Physical Therapy, Universidade Federal de Sergipe (UFS), São Cristóvão, Sergipe, Brazil.; 2 Post-Graduation Program in Health Sciences, Universidade Federal de Sergipe (UFS), São Cristóvão, Sergipe, Brazil.; 3 The GREAT Group (GRupo de Estudos em ATividade física), Universidade Federal de Sergipe (UFS), São Cristóvão, Sergipe, Brazil.; 4 Investigative Pathology Laboratory, Universidade Federal de Sergipe (UFS), São Cristóvão, Sergipe, Brazil.; 5 Department of Physical Therapy, Universidade Federal da Bahia (UFBa), Salvador, Bahia, Brazil.; 6 Universidade de Pernambuco (UPE), Pernambuco, Recife, Brazil.

**Keywords:** Heart Transplantation, High-Intensity Interval Training, Exercise Tolerance, Heart Failure

## Abstract

**Introduction:**

Heart transplantation (HTx) is the gold standard procedure for selected individuals with refractory heart failure. High-intensity interval training (HIIT) is safe and allows patients to exercise in high intensity for longer time when compared to moderate-intensity continuous training (MICT). The primary aim of this study was to perform a systematic review and meta-analysis about the effect of HIIT compared to MICT on exercise capacity, peak heart rate, and heart rate reserve in HTx recipients. Secondarily, we pooled data comparing MICT and no exercise training in these patients.

**Methods:**

This systematic review followed the standardization of the Preferred Reporting Items for Systematic Reviews and Meta-analyses statement and the Cochrane Collaboration Handbook. We presented the treatment effects of HIIT on the outcomes of interest as mean difference (MD) and 95% confidence interval (CI). Meta-analysis was performed using the random-effects, generic inverse variance method.

**Results:**

HIIT improved peak oxygen consumption (peakVO2) (MD = 2.1; 95% CI 1.1, 3.1; *P*<0.0001), peak heart rate (MD = 3.4; 95% CI 0.8, 5.9; *P*=0.009), and heart rate reserve (MD = 4.8; 95% CI -0.05, 9.6; *P*=0.05) compared to MICT. Improvements on peakVO_2_ (MD = 3.5; 95% CI 2.3, 4.7; *P*<0.00001) and peak heart rate (MD = 5.6; 95% CI 1.6, 9.6; *P*=0.006) were found comparing HIIT and no exercise training.

**Conclusion:**

Current available evidence suggests that HIIT leads to improvements on peakVO_2_, peak heart rate, and heart rate reserve compared to MICT in HTx recipients. However, the superiority of HIIT should be tested in isocaloric protocols.

**Table:** 

Abbreviations, acronyms & symbols				
**ACT**	**= Active recovery**		**MD**	**= Mean difference**
**AIT**	**= Aerobic interval training**		**MICT**	**= Moderate-intensity continuous training**
**CI**	**= Confidence interval**		**NR**	**= Not reported**
**F**	**= Female**		**PeakVO_2_**	**= Peak oxygen consumption**
**HIIT**	**= High-intensity interval training**		**RCTs**	**= Randomized controlled trials**
**HR**	**= Hear rate**		**SD**	**= Standard deviation**
**HRQoL**	**= Health-related quality of life**		**SDc**	**= Standard deviation of change**
**HTx**	**= Heart transplantation**		**SE**	**= Standard error**
**M**	**= Male**			

## INTRODUCTION

Heart transplantation (HTx) is the gold standard procedure for selected individuals with end-stage heart failure^[1]^. Even knowing that HTx improves patients’ exercise tolerance, it is not restored to normal values^[2]^. The concern about exercise tolerance in HTx recipients is based on the association between peak oxygen consumption (peakVO_2_), the gold standard method to assess cardiorespiratory fitness, and survival^[3,4]^.

Several studies have demonstrated the positive effects of aerobic exercise training on peakVO_2_ in HTx recipients^[5-7]^. However, there is no consensus on how, when, or at what intensity exercise should be performed by HTx patients^[8]^. High-intensity interval training (HIIT) allows patients to exercise in higher intensity when compared to standard continuous training. A previous well performed meta-analysis^[9]^ showed that HIIT was safe and effective in improving peakVO_2_ in HTx recipients when compared to no training. However, the lack of studies that compared HIIT with moderate-intensity continuous training (MICT) limited the previous meta-analysis. Moreover, conflicting results about HIIT superiority are still under discussion in cardiovascular rehabilitation^[10,11]^.

Since the previous meta-analysis was published^[9]^, new randomized controlled trials (RCTs) comparing HIIT with MICT were published^[12,13]^. Due to the new available data, the primary aim of the present study was to perform a systematic review and meta-analysis to synthesize evidence about the effect of HIIT compared to MICT on exercise capacity, peak heart rate, and heart rate reserve in HTx recipients. Secondarily, we pool data from trials comparing HIIT and no exercise training in these patients.

## METHODS

**Fig. 1:**
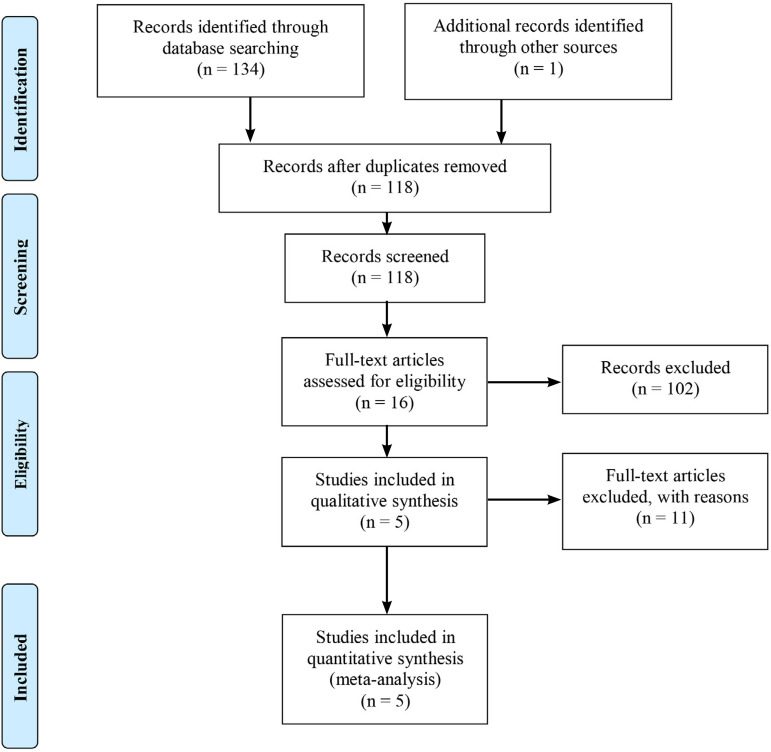
Flow diagram showing the reference screening and study selection

This study was conducted following the Preferred Reporting Items for Systematic Reviews and Meta-analyses statement^[14]^ and supplemented by guidance from the Cochrane Collaboration Handbook for Systematic Reviews of Interventions^[15]^. A flow diagram showing the reference screening and study selection is presented on Figure 1.

### Eligibility Criteria

To establish the eligibility criteria we used the following PICOT elements (standing for Participants, Intervention, Comparison, Outcomes, and Study type): *Population*, HTx recipients; *Intervention*, HIIT; *Comparison*, MICT or no exercise training; *Primary outcome*, peakVO_2_ (ml/kg/min); *Secondary outcomes*, peak heart rate and heart rate reserve (bpm); *Study type*, RCTs.

### Search Strategy

We searched for references in PubMed, Scopus, and Cochrane Central Register of Controlled Trials up to February 2019. A gray literature search included Google Scholar and OpenThesis. The first 100 results of the Google Scholar search were analyzed. The search was limited to studies published in full-text versions, without language restriction. Search strategy is provided in the online supplement. The reference lists of all eligible studies and reviews were also scanned to identify additional studies for inclusion. The authors were contact by e-mail for confirmation of any data or additional information if needed.

### Quality of the Studies

**Fig. 2:**
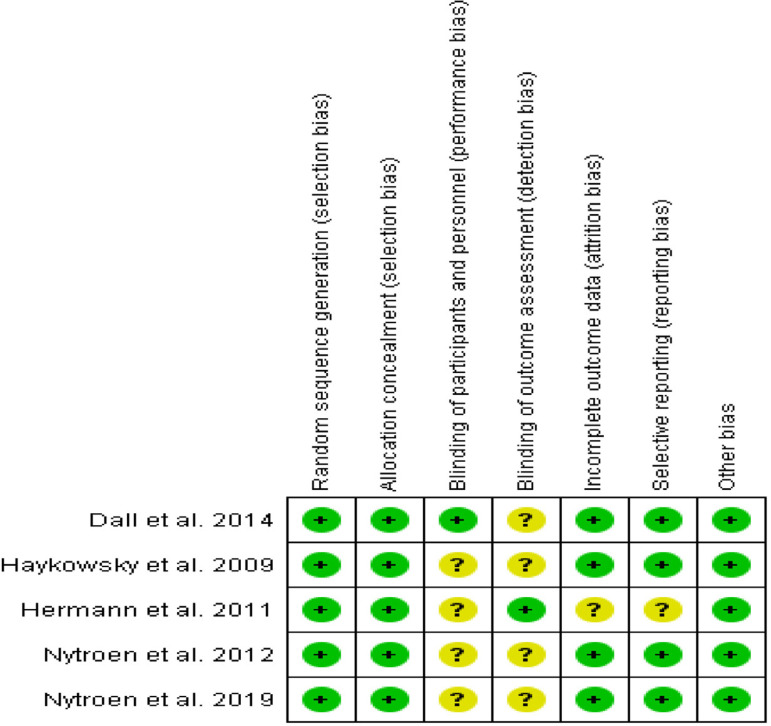
Risk of bias summary: review authors' assessments about each risk of bias item for each included study.

**Fig. 3:**
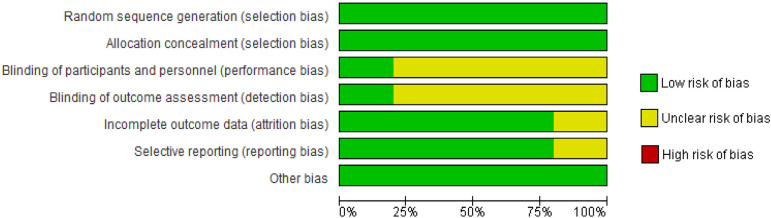
Risk of bias graph: review authors' assessments about each risk of bias item presented as percentages across all included studies

Risk of bias was assessed according to the Cochrane guidelines for RCTs. Seven domains were assessed for evaluation: sequence generation and allocation concealment (selection bias), blinding of participants and personnel (performance bias), blinding of outcome assessment (detection bias), incomplete outcome data (attrition bias), selective outcome reporting (reporting bias), and other potential sources of bias (Figure 2). Risk of bias was rated as low, unclear, or high according to established criteria (Figure 3)^[16]^.

### Data Collection and Analysis

Two independent reviewers (COG and RESF) searched for relevant studies according to title and abstract. If at least one of the reviewers considered one reference eligible, the full text was obtained for a complete assessment. Then, the two reviewers assessed the full text according to the eligibility criteria. Thereafter, the following information from the studies were extracted: demographic characteristics of study participants, exercise protocols, adverse events, and outcome data. Values (mean difference [MD] and standard deviation [SD]) for peakVO_2_, peak heart rate, and heart rate reserve were extracted before and after intervention.

The meta-analysis was performed using the change between pre- and post-intervention means for each intervention group and the change SD calculated. If the SD of change [SDc]) for a given outcome was not reported, the formula:

$\mathrm{SDc}=\sqrt{\left[{\left({\mathrm{SD}}_{\mathrm{pre}}\right)}^{2}+{\left({\mathrm{SD}}_{\mathrm{post}}\right)}^{2}-\left(2\times {\mathrm{corr}}_{\mathrm{pre},\mathrm{post}}\times {\mathrm{SD}}_{\mathrm{pre}}\times {\mathrm{SD}}_{\mathrm{post}}\right)\right]}$

was applied. SD_pre_, SD_post_, and corr_pre,post_ represent the SD of the pre-intervention value, the SD of the post-intervention value, and the correlation coefficient between pre- and post-intervention values, respectively. The corr_pre,post_ was conservatively set at 0.5. Mean changes were pooled using the random-effects, generic inverse variance method. A forest plot was used to graphically present the effect sizes and the 95% confidence intervals (CIs). A two-tailed *P*-value of <0.05 was used to determine significance. Statistical heterogeneity was assessed by using the Cochran Q test^[17]^ and quantified by the I^2^ index^[18]^. We conducted all analyses using Review Manager 5.3 (Cochrane IMS, Copenhagen, Denmark).

## RESULTS

### Studies Characteristics

Five RCTs^[5,12,13]^ (total of 212 HTx recipients with mean age of 57 years) were included in this systematic review. Exercise protocols were well reported (Tables 1 and 2). Follow-up periods were also well reported among studies: eight weeks^[17,18]^, 12 weeks^[19,20]^, and one year^[8]^. No serious adverse events were reported by the studies. In general, the studies presented a low/uncertain risk of bias. Figure 3 presents results of individual assessment by Cochrane risk of bias.

**Table 1:** Characteristics of included studies.

Study	Disease	Sample size (including dropouts)	Gender	Age (years)	Time after transplantation	Outcomes	Key findings	Dropouts (%)	Dropouts before and after intervention
Nytrøen et al.^[8]^, 2019	HTx	81	M (66)/F (15)	50	3 months	PeakVO_2_, HRQoL, left ventricular function, endothelial function, and biomarkers	HIIT group demonstrated greater improvements than those observed in the MICT group (mean difference: 1.8 ml/kg/min)	3.8%	3.8%
Dall et al.^[13]^, 2014	HTx (normal chronotropic response and chronotropic impairment)	20 (16)	M (12)/F (4)	51.9	6.4 years	PeakVO_2_, blood pressure, HRpeak, HRrest, HRreserve, HRrecovery, workload	There was an improve in peakVO_2_ (0,001), HRpeak (0,014), and HRreserve (0,012)	All - 3 (20%)	It was a crossover study. NR
Nytrøen et al.^[18]^, 2012	HTx	57 (52)	M (33)/F (15)	51	4.1 years	Exercise capacity (peakVO_2_ and peakVO_2_ predicted), body composition, biochemistry, and HRQoL	HIIT improved peakVO_2_ (0.001), HRreserve (0,002), HRpeak (0.035)	All - 9 (15%)	HIIt - 2 (8%), control group - 2 (8%)
Hermann et al.^[19]^, 2011	HTx	30 (27)	M (22)/F (5)	50	6,9 years	PeakVO_2_, endothelial function, blood pressure, markers of inflammation	PeakVO_2_ was higher in the HIIT group (0,001), flow-mediated vasodilation (0,048), reduced blood systolic pressure (0,03), reduced plasma levels (0,02)	All - 3 (10%)	HIIT - 2 (6,7%), control group - 1 (3,3%)

F=female; HIIT=high-intensity interval training; HR=heart rate; HRQoL=health-related quality of life; HTx=heart transplantation; M=male; MICT=moderateintensity continuous training; NR=not reported; peakVO_2_=peak oxygen consumption

**Table 2:** Characteristics of the HIIT vs. MICT intervention in the trials included in the review.

Study	Type of exercise	VO_2_measurement	Intensity	Trained intensity	Volume	Frequency (´ per week)	Length (weeks)	Supervision
Nytrøen et al.^[8]^, 2019	HIIT (AIT)	Cardiopulmonary test	81-93% peak effort	10 min warm-up4 bouts (4 min of HIIT) and 3 bouts (3 min of MICT)5 min cooldown	3	02/mar	39	Nytrøen et al., 2019
	MICT (ACT)	Cardiopulmonary test	60-80% peak effort	10 min warm-up	3	02/mar	39	
				25 min exercise				
				5 min cooldown				
Dall et al.^[13]^, 2014	HIIT (AIT)	Cardiopulmonary test	> 80%peakVO_2_	10 min warm-up	3	3	12	Dall et al., 2014
				16 min exercise				
				10 min cooldown				
	MICT (ACT)	Cardiopulmonary test	60-70% peakVO_2_	10 min warm-up	3	3	12	
				45 min exercise				
				10 min cooldown				
Hermann et al.^[19]^, 2011	HIITa	Cardiopulmonary test	80%, 85%, and 90% of peakVO_2_	10 min warm-up	3	3	8	Hermann et al., 2011
				42 min exercise				
				10 min cooldown				
	Control group (sedentary?) Only education	Cardiopulmonary test	NR	NR	NR	NR	NR	
Nytrøen et al.^[18]^, 2012	HIIT	Cardiopulmonary test	85-95% HRmax	10 min warm-up	3	3	8	Nytrøen et al., 2012
				16 min exercise				
				NR cooldown				
	Control group (no intervention)	Cardiopulmonary test	NR	NR	NR	NR	NR	
Haykowsky et al.^[5]^, 2009	HIIT	Cardiopulmonary test	60-80% peakVO_2_	10 min warm-up	3	5´/week (12 weeks)	12	Nytrøen et al., 2013
				16 min exercise				
				NR cooldown				
	Control group (no intervention)	Cardiopulmonary test	NR	NR	NR	NR	NR	

ACT=active recovery; AIT=aerobic interval training; HIIT=high-intensity interval training; HR=heart rate; MICT=moderate-intensity continuous training; NR=Not reported

### Overall Analysis

**Fig. 4:**
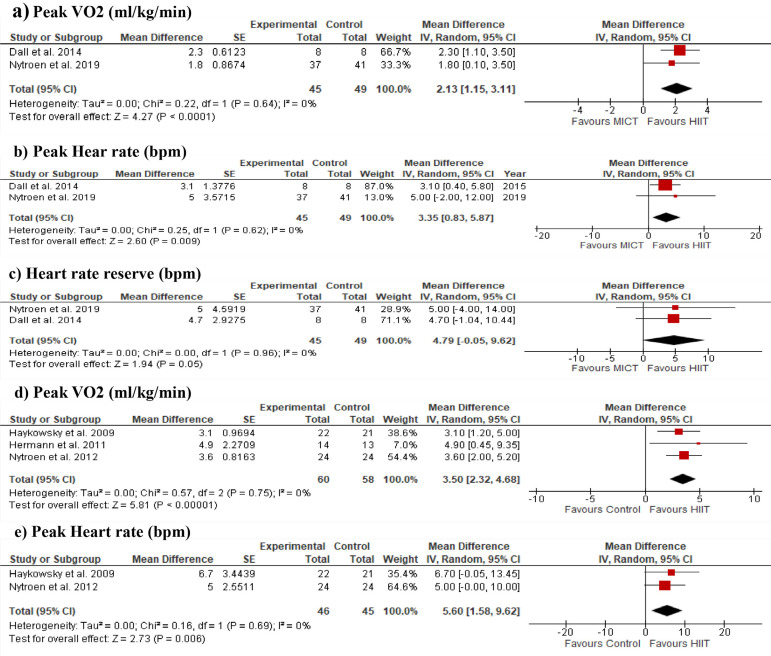
Forest plot showing meta-analyses of the high-intensity interval training (HIIT) vs. moderate-intensity continuous training (MICT) on a) peak oxygen consumption (peakVO2), b) peak heart rate, and c) heart rate reserve. Comparisons between HIIT vs. no exercise training on d) peakVO2 and e) peak heart rate were also shown. CI=confidence interval; SE=standard error

HIIT improved peakVO_2_ (MD = 2.1; 95% CI 1.1, 3.1; *P*<0.0001) (Figure 4A), peak heart rate (MD = 3.4; 95% CI 0.8, 5.9; *P*=0.009) (Figure 4B), and heart rate reserve (MD = 4.8; 95% CI -0.05, 9.6; *P*=0.05) (Figure 4C) compared to MICT. Improvements on peakVO_2_ (MD = 3.5; 95% CI 2.3, 4.7; *P*<0.00001) (Figure 4D) and peak heart rate (MD = 5.6; 95% CI 1.6, 9.6; *P*=0.006) (Figure 4E) were also found comparing HIIT and no exercise training. No data was available to compare heart rate reserve between HIIT and control without exercise. No between-study heterogeneity (I^2^ = 0%) was found in the meta-analyses.

## DISCUSSION

Our systematic review found that HIIT was superior than MICT on peakVO_2_, peak heart rate, and heart rate reserve in HTx recipients. In recent years, a growing number of studies have suggested that HIIT is similar or even superior to MICT in peakVO_2_, heart rate response, and quality of life improvements^[8,13,21]^.

A previous meta-analysis reported the efficiency of HIIT on peakVO_2_ gains in HTx when compared to no exercise training^[9]^. However, our meta-analysis contributes to a better understanding of the effect size of HIIT when compared with the standard exercise intensity prescription (MICT). Additionally, some issues regarding the effects of exercise protocols must be addressed in relation to the apparent superiority of HIIT over MICT. In a recent review, Dun et al.^[22]^ argued that the exercise duration and ratio of high and low-intensity bouts are key factors that differentiate HIIT from MICT and may contribute to the patient's response. In general there are three main protocols of HIIT: long, medium, and short. The long-duration HIIT protocol (four minutes at high intensity - 85-95% peakVO_2_ - and three minutes at moderate intensity - 60-70% of peakVO_2_) is the most widely used in patients with cardiovascular diseases. Another meta-analysis demonstrated that the long-duration HIIT protocols were associated with larger improvements in peakVO_2_ in healthy individuals^[23]^. In our meta-analysis, the studies comparing HIIT to MICT used long-duration HIIT protocol. This may be one of the main factors behind the superiority of HIIT over MICT^[22]^.

The concept of isocaloric protocol must also be considered when HIIT and MICT are compared. The superiority of HIIT over MICT disappeared when studies that used isocaloric protocols were analyzed in previous meta-analyses involving patients with coronary artery disease^[11]^ and heart failure^[10]^. In our analysis, only one study^[23]^ reported isocaloric protocol, which limits any pragmatic conclusion about HIIT superiority.

Additionally, our secondary analysis demonstrated that HIIT had superior effects to MICT in peak heart rate and heart rate reserve. In four of the five included studies, the mean time after transplantation was between four and six years^[13,16-18]^. Initially, we supposed that this superiority would be associated with cardiac reinnervation. Long-term HTx recipients are expected to show some degree of cardiac reinnervation, which nearly normalizes heart rate control. In contrast, newly HTx recipients display a denervated status and the heart rate response is markedly reduced compared to health individuals. On the other hand, in one study, HTx recipients were followed 8-12 weeks after HTx^[12]^. The apparent superiority of HIIT may be associated with the volume of training^[23]^. Additionally, the small number of studies and of isocaloric protocols does not allow us to fully support the superiority of HIIT over MICT in cardiac dynamics.

### Limitation

Caution is warranted in interpreting the results of this study. One important limitation is the low number of studies comparing HIIT to MICT and the lack of isocaloric protocols. Another important limitation is that in just one study^[13]^ the patients were followed by a physical therapist in a 1:1 setting. This type of setting may guarantee that the patients reach the proper intensity prescribed. On the other hand, this type of setting may not be suitable in most cardiac rehabilitation centers around the world, especially in middle- and lower-income countries.

## CONCLUSION

In conclusion, current available evidence suggests that HIIT is superior to MICT on peakVO_2_, peak heart rate, and heart rate reserve improvements in HTx recipients. However, new RCTs are necessary to analyze the influence of isocaloric protocols and different duration protocols on peakVO_2_ in HTx recipients.

**Table:** 

Authors' roles & responsibilities	
LSRC	Drafting the work; final approval of the version to be published; leading author
COG	Substantial contributions to the acquisition of data for the work; final approval of the version to be published
RESF	Substantial contributions to the acquisition of data for the work; final approval of the version to be published
PRSMF	Statistical analysis and Substantial contributions to the conception or design of the work.
MGN	Substantial contributions to the analysis of data for the work; revising the work; final approval of the version to be published
VRN	Drafting the work and revising it; final approval of the version to be published
VOC	Substantial contributions to the acquisition of data for the work; drafting the work; final approval of the version to be published

## Supplementary Content

**Search Strategy:**

**PubMed:**

("heart recipient" OR "heart transplant recipient" OR "heart transplant" OR "cardiac transplant" OR "heart graft" OR "heart transplantation"[Mesh] OR "cardiac transplantation") AND ("exercise training" OR "interval training" OR "high intensity interval training" OR "high intensity training" OR "intermittent training") OR "sprint training") AND (randomized controlled trial[Publication Type] OR (randomized[Title/Abstract] AND controlled[Title/Abstract] AND trial[Title/Abstract])

**Scopus:**

("Heart Transplantation" OR "Heart Grafting" OR "Cardiac Transplantation" OR "cardiac transplantations") AND ("High Intensity Interval Training" OR "Sprint Interval Training" OR "Sprint Interval Trainings" OR "High-Intensity Intermittent Exercise")

**Cochrane Library:**

("Heart Transplantation" [Mesh term]) AND ("High-Intensity Interval Training"[Mesh term])
